# Rhythmotherapy, or a Discussion of the Physiologic Basis and Therapeutic Potency of Mechano-Vital Vibration

**Published:** 1907-08

**Authors:** 


**Rhythmotherapy, or a Discussion of the Physiologic Basis and Therapeutic Potency of Mechano-vital Vibration;** to which is added a dictionary of Diseases. By **Samuel S. Wallian,** A.M., M.D. Chicago: Ouellette Press. 1906. (Price, $1.50).

This is one of those rare bits of harlequin literature which, while garlanded with smooth phrase and lop-lidded description in an apparent effort to disguise it as a pseudo scientific bit of gleaning is, stripped of its finery and laid bare to the cold and calculating gaze of a coarse and unsympathetic reading people, little more than a business boost for a series of business concerns which are doing quite well, thank you, giving a large number of ill and near-ill people an assorted series of finely regulated jiggles. If one goes to a physician who has listened long enough to the human encyclopedias who sell them and has installed a machine in his office, the patient gets a rythmotherapeutic treatment; if the gentleman with the Willies or the lady with a knobby knee buys a fifty cent apparatus at a drug store, they take vibration themselves. The book announces itself modestly as a discussion of the physiologic basis and therapeutic potency of mechanovital vibration, to which is added a dictionary of diseases with suggestions as to the technic of vibratory therapeutics and it is "by" Samuel S. Wallian, A.M., M.D., who is bannered by the following personal and professional description on the title page: "President American Medico-Pharmaceutical League; ex-President Medical Association of Northern New York; Member of New York State and County Medical Societies; Fellow of the American Electro-Therapeutic Association; Member Medico-Legal Society; Associate Editor, Medico-Pharmaceutical Journal, Etc."

The book is copyrighted and all rights are reserved which was an unnecessary expense on the part of the author.

It may appear to the reader that an extended review of this delicate brochure is a waste of space; but there are occasions when it is merciful to wake up the unconscious victim of autohpynosis before he walks off the roof.

The author and member soars to heights which are beyond the ken of we poor mortals who have not learned to gaze into the crystal globe of vibratory vagaries and gives up a page of fair, white paper to such ultramarine tommyrot as "the morning stars have been singing together since the primal dawn:" "Life is an incessant succession of rythmic reiteration;" "Disease is an opening of a discordant stop;" "Death is a cessation of vibrant impulse. It shuts the organ manual:" "The 'Voice of Nature' is the diapason of the Infinite." And while up in the air among those sublime heights where poets dream, the member must have plucked a couple of blooms from someone's garden, for he adds: "Music is love struggling for a language;" "Love is music that has found a language."

There are many pages of argument and excuse and of megaphonic commendation of the new rythmotherapy; there is a careless and familiar tossing about of gems of thought concerning cell and creation, and then there is the grand dive from the top of the tent into the net and we are improved by having revealed to us the mysteries of the dictionary of diseases.

The reviewer, with youth and an open heart on his side, writes not in condemnation of vibratory treatment; he merely calls attention in simple language to the fool things which may happen in literature when printing is cheap and the various little worlds in which we live go spinning about light footed with prosperity and heavy topped with fads.

A few of the diseases and some of the ideas from the dictionary will be of assistance to the young practitioner in guiding his footsteps in the right path and showing him what not to do.

The *x*-ray and radium may and undoubtedly will do good in carcinoma as they have in lupus; but "if vibration had been used" the failures of these comparatively new agencies might have been successes. The diseased appendix is vibrated and threatened explosions may be averted; dermoids may be dispersed by vibration; degenerations such as tabes, marasmum and tuberculosis fall beneath the onward march of the vibrator; gallstones are as putty in the hands of the vibratist—all he has to do to manipulate the liver until the duct relaxes and the stones pass; movable kidney is a mere incident to the Member, for while deprecating the fact that such cases are too frequently "turned over to the tender mercy of the surgeon's knife," he cures "many cases without resort to this dreaded expedient." Locomotor ataxia, and leucorrhea travel hand in hand in the ranks of the never-agains. Spinal applications are recommended for renal calculus as well as direct application over the site of the kidney, which may possibly be all right for a patient who has healthy kidneys, but there is an element of personal danger in trying it on a man with a renal calculus, especially if he be one of those large, coarse and muscular persons susceptible to pain. There is some good in the book; candor and a congenial tendency to truthfulness compels this statement even though it refers to the excellence of the workmanship of the half-tone plates and the colored plates showing nerve distribution, one of which portrays a gentleman patiently holding the Delsartian attitude of despair and who is gaily decorated with a fine centerpiece representing a fig leaf, which one less botanically acute than the writer might in a misguided moment of hasty conclusion take to be a sprig of poison ivy.

A clever writer satirising present day politics had one of his characters remark: "For every little good in the world there is a deal of that's no use." As for the book itself, its chief fault is its fanatic attitude; its chief virtue, is its size—it is very small.

